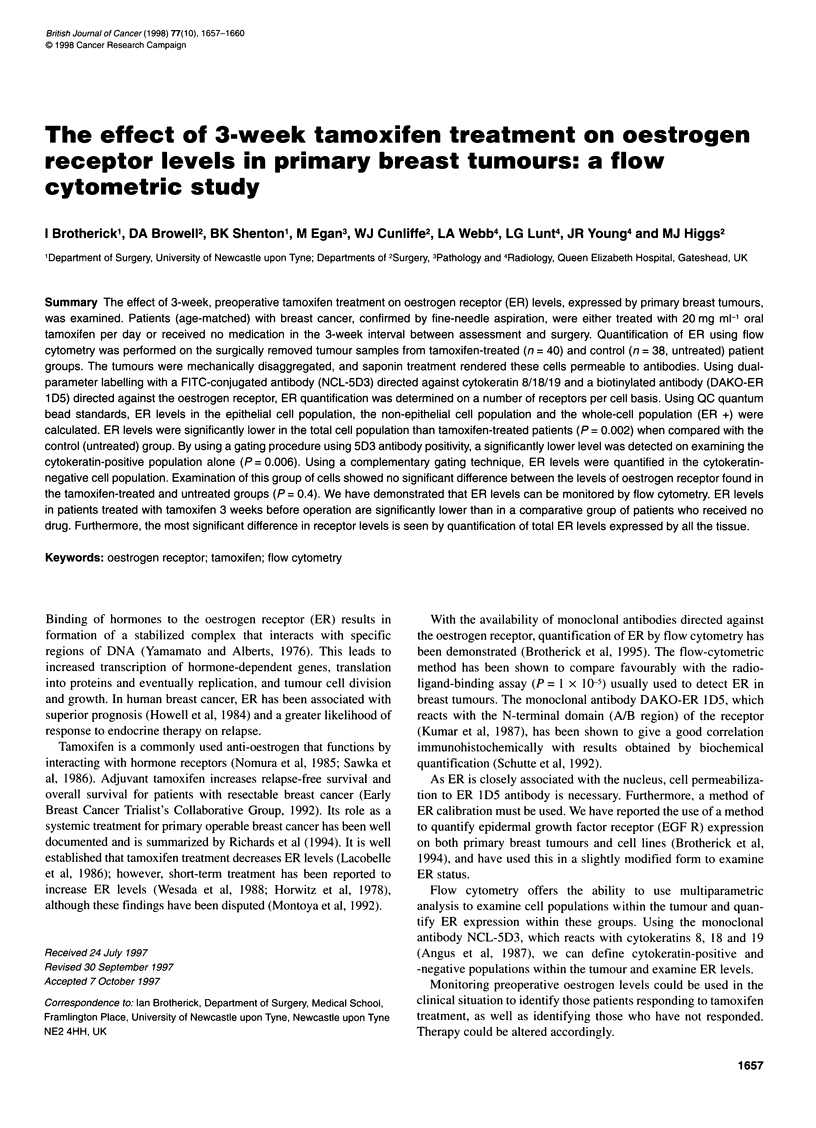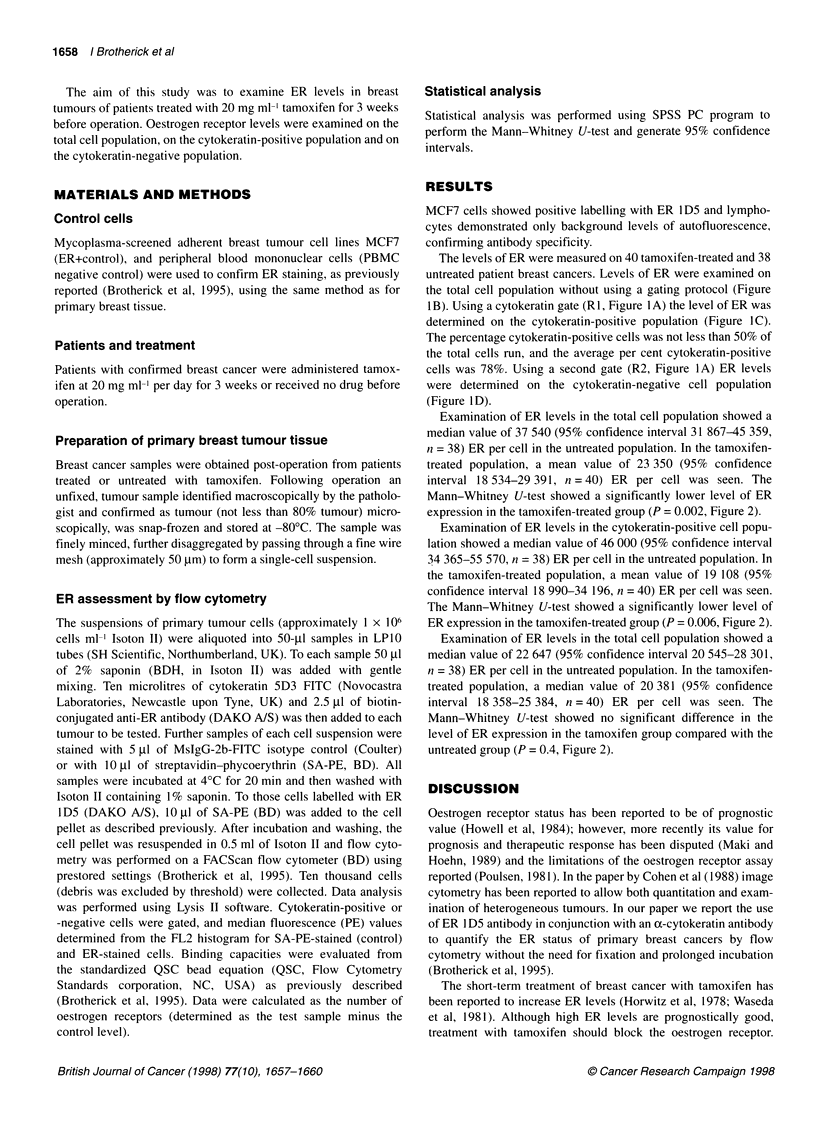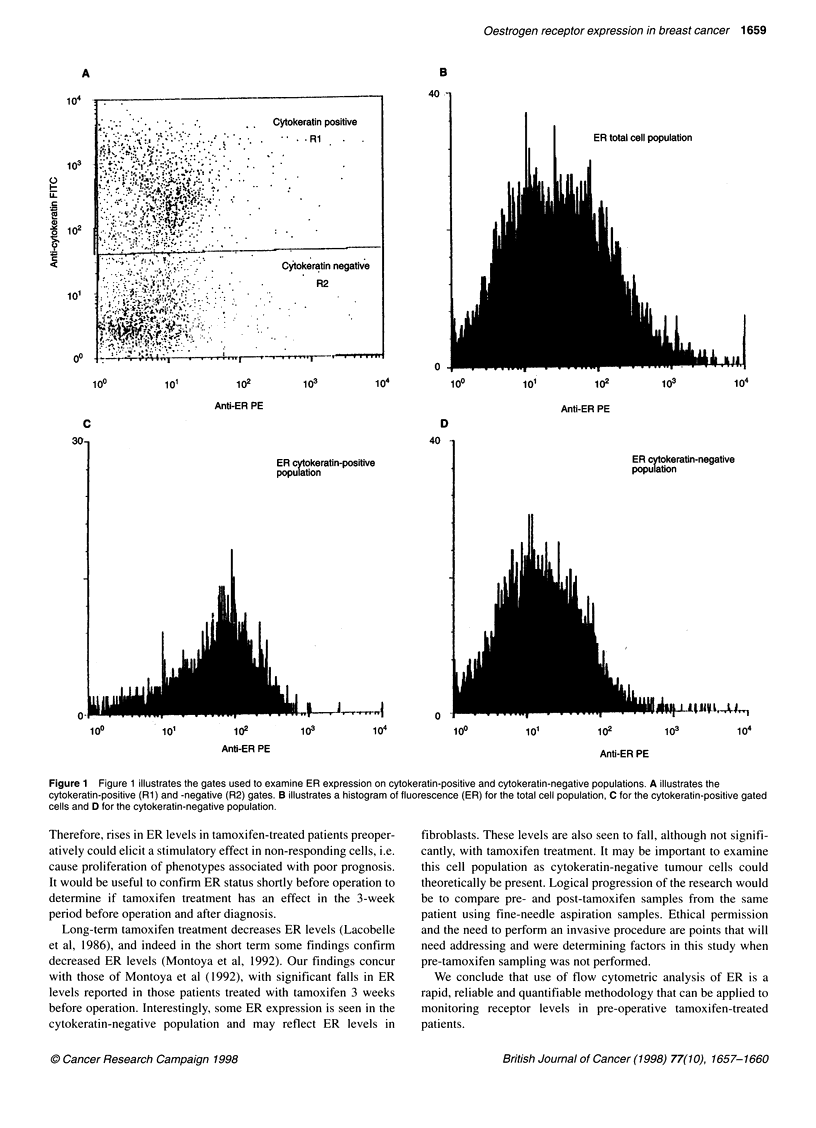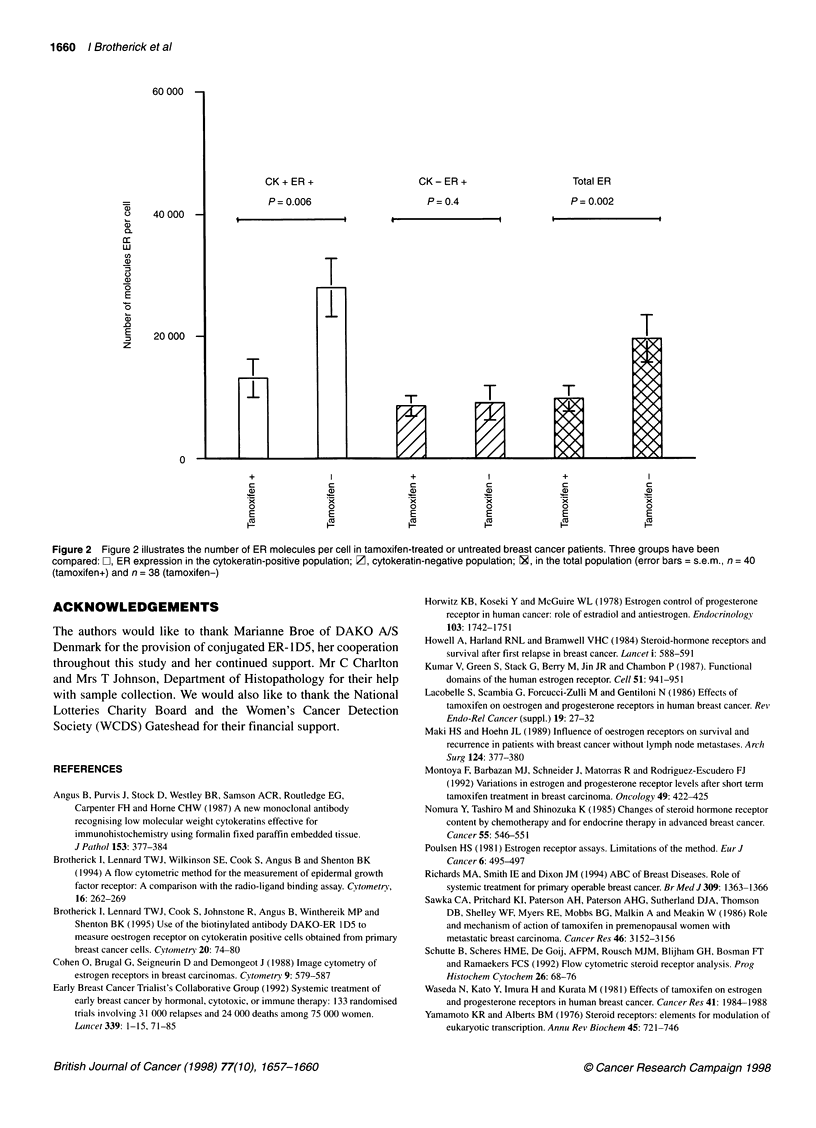# The effect of 3-week tamoxifen treatment on oestrogen receptor levels in primary breast tumours: a flow cytometric study.

**DOI:** 10.1038/bjc.1998.272

**Published:** 1998-05

**Authors:** I. Brotherick, D. A. Browell, B. K. Shenton, M. Egan, W. J. Cunliffe, L. A. Webb, L. G. Lunt, J. R. Young, M. J. Higgs

**Affiliations:** Department of Surgery, University of Newcastle upon Tyne, UK.

## Abstract

The effect of 3-week, preoperative tamoxifen treatment on oestrogen receptor (ER) levels, expressed by primary breast tumours, was examined. Patients (age-matched) with breast cancer, confirmed by fine-needle aspiration, were either treated with 20 mg ml(-1) oral tamoxifen per day or received no medication in the 3-week interval between assessment and surgery. Quantification of ER using flow cytometry was performed on the surgically removed tumour samples from tamoxifen-treated (n = 40) and control (n = 38, untreated) patient groups. The tumours were mechanically disaggregated, and saponin treatment rendered these cells permeable to antibodies. Using dual-parameter labelling with a FITC-conjugated antibody (NCL-5D3) directed against cytokeratin 8/18/19 and a biotinylated antibody (DAKO-ER 1D5) directed against the oestrogen receptor, ER quantification was determined on a number of receptors per cell basis. Using QC quantum bead standards, ER levels in the epithelial cell population, the non-epithelial cell population and the whole-cell population (ER+) were calculated. ER levels were significantly lower in the total cell population than tamoxifen-treated patients (P = 0.002) when compared with the control (untreated) group. By using a gating procedure using 5D3 antibody positivity, a significantly lower level was detected on examining the cytokeratin-positive population alone (P = 0.006). Using a complementary gating technique, ER levels were quantified in the cytokeratin-negative cell population. Examination of this group of cells showed no significant difference between the levels of oestrogen receptor found in the tamoxifen-treated and untreated groups (P = 0.4). We have demonstrated that ER levels can be monitored by flow cytometry. ER levels in patients treated with tamoxifen 3 weeks before operation are significantly lower than in a comparative group of patients who received no drug. Furthermore, the most significant difference in receptor levels is seen by quantification of total ER levels expressed by all the tissue.


British Journal of Cancer (1998) 77(10), 1657–1660
© 1998 Cancer Research Campaign

### The effect of 3-week tamoxifen treatment on oestrogen receptor levels in primary breast tumours: a flow cytometric study

**I Brotherick[1], DA Browell[2], BK Shenton[1], M Egan[3], WJ Cunliffe[2], LA Webb[4], LG Lunt[4], JR Young[4] and MJ Higgs[2]**

[1]Department of Surgery, University of Newcastle upon Tyne; Departments of [2]Surgery, [3]Pathology and [4]Radiology, Queen Elizabeth Hospital, Gateshead, UK

**Summary** The effect of 3-week, preoperative tamoxifen treatment on oestrogen receptor (ER) levels, expressed by primary breast tumours, was examined. Patients (age-matched) with breast cancer, confirmed by fine-needle aspiration, were either treated with 20 mg $ml^{-1}$ oral tamoxifen per day or received no medication in the 3-week interval between assessment and surgery. Quantification of ER using flow cytometry was performed on the surgically removed tumour samples from tamoxifen-treated ($n = 40$) and control ($n = 38$, untreated) patient groups. The tumours were mechanically disaggregated, and saponin treatment rendered these cells permeable to antibodies. Using dual-parameter labelling with a FITC-conjugated antibody (NCL-5D3) directed against cytokeratin 8/18/19 and a biotinylated antibody (DAKO-ER 1D5) directed against the oestrogen receptor, ER quantification was determined on a number of receptors per cell basis. Using QC quantum bead standards, ER levels in the epithelial cell population, the non-epithelial cell population and the whole-cell population (ER +) were calculated. ER levels were significantly lower in the total cell population than tamoxifen-treated patients ($P = 0.002$) when compared with the control (untreated) group. By using a gating procedure using 5D3 antibody positivity, a significantly lower level was detected on examining the cytokeratin-positive population alone ($P = 0.006$). Using a complementary gating technique, ER levels were quantified in the cytokeratin-negative cell population. Examination of this group of cells showed no significant difference between the levels of oestrogen receptor found in the tamoxifen-treated and untreated groups ($P = 0.4$). We have demonstrated that ER levels can be monitored by flow cytometry. ER levels in patients treated with tamoxifen 3 weeks before operation are significantly lower than in a comparative group of patients who received no drug. Furthermore, the most significant difference in receptor levels is seen by quantification of total ER levels expressed by all the tissue.

**Keywords:** oestrogen receptor; tamoxifen; flow cytometry

Binding of hormones to the oestrogen receptor (ER) results in formation of a stabilized complex that interacts with specific regions of DNA (Yamamato and Alberts, 1976). This leads to increased transcription of hormone-dependent genes, translation into proteins and eventually replication, and tumour cell division and growth. In human breast cancer, ER has been associated with superior prognosis (Howell et al, 1984) and a greater likelihood of response to endocrine therapy on relapse.

Tamoxifen is a commonly used anti-oestrogen that functions by interacting with hormone receptors (Nomura et al, 1985; Sawka et al, 1986). Adjuvant tamoxifen increases relapse-free survival and overall survival for patients with resectable breast cancer (Early Breast Cancer Trialist's Collaborative Group, 1992). Its role as a systemic treatment for primary operable breast cancer has been well documented and is summarized by Richards et al (1994). It is well established that tamoxifen treatment decreases ER levels (Lacobelle et al, 1986); however, short-term treatment has been reported to increase ER levels (Wesada et al, 1988; Horwitz et al, 1978), although these findings have been disputed (Montoya et al, 1992).

*Received 24 July 1997*
*Revised 30 September 1997*
*Accepted 7 October 1997*

*Correspondence to:* Ian Brotherick, Department of Surgery, Medical School, Framlington Place, University of Newcastle upon Tyne, Newcastle upon Tyne NE2 4HH, UK

With the availability of monoclonal antibodies directed against the oestrogen receptor, quantification of ER by flow cytometry has been demonstrated (Brotherick et al, 1995). The flow-cytometric method has been shown to compare favourably with the radio-ligand-binding assay ($P = 1 \times 10^{-5}$) usually used to detect ER in breast tumours. The monoclonal antibody DAKO-ER 1D5, which reacts with the N-terminal domain (A/B region) of the receptor (Kumar et al, 1987), has been shown to give a good correlation immunohistochemically with results obtained by biochemical quantification (Schutte et al, 1992).

As ER is closely associated with the nucleus, cell permeabilization to ER 1D5 antibody is necessary. Furthermore, a method of ER calibration must be used. We have reported the use of a method to quantify epidermal growth factor receptor (EGF R) expression on both primary breast tumours and cell lines (Brotherick et al, 1994), and have used this in a slightly modified form to examine ER status.

Flow cytometry offers the ability to use multiparametric analysis to examine cell populations within the tumour and quantify ER expression within these groups. Using the monoclonal antibody NCL-5D3, which reacts with cytokeratins 8, 18 and 19 (Angus et al, 1987), we can define cytokeratin-positive and -negative populations within the tumour and examine ER levels.

Monitoring preoperative oestrogen levels could be used in the clinical situation to identify those patients responding to tamoxifen treatment, as well as identifying those who have not responded. Therapy could be altered accordingly.

The aim of this study was to examine ER levels in breast tumours of patients treated with 20 mg ml$^{-1}$ tamoxifen for 3 weeks before operation. Oestrogen receptor levels were examined on the total cell population, on the cytokeratin-positive population and on the cytokeratin-negative population.

### MATERIALS AND METHODS

### Control cells

Mycoplasma-screened adherent breast tumour cell lines MCF7 (ER+control), and peripheral blood mononuclear cells (PBMC negative control) were used to confirm ER staining, as previously reported (Brotherick et al, 1995), using the same method as for primary breast tissue.

### Patients and treatment

Patients with confirmed breast cancer were administered tamoxifen at 20 mg ml$^{-1}$ per day for 3 weeks or received no drug before operation.

### Preparation of primary breast tumour tissue

Breast cancer samples were obtained post-operation from patients treated or untreated with tamoxifen. Following operation an unfixed, tumour sample identified macroscopically by the pathologist and confirmed as tumour (not less than 80% tumour) microscopically, was snap-frozen and stored at –80°C. The sample was finely minced, further disaggregated by passing through a fine wire mesh (approximately 50 µm) to form a single-cell suspension.

### ER assessment by flow cytometry

The suspensions of primary tumour cells (approximately $1 \times 10^6$ cells ml$^{-1}$ Isoton II) were aliquoted into 50-µl samples in LP10 tubes (SH Scientific, Northumberland, UK). To each sample 50 µl of 2% saponin (BDH, in Isoton II) was added with gentle mixing. Ten microlitres of cytokeratin 5D3 FITC (Novocastra Laboratories, Newcastle upon Tyne, UK) and 2.5 µl of biotin-conjugated anti-ER antibody (DAKO A/S) was then added to each tumour to be tested. Further samples of each cell suspension were stained with 5 µl of MsIgG-2b-FITC isotype control (Coulter) or with 10 µl of streptavidin–phycoerythrin (SA-PE, BD). All samples were incubated at 4°C for 20 min and then washed with Isoton II containing 1% saponin. To those cells labelled with ER 1D5 (DAKO A/S), 10 µl of SA-PE (BD) was added to the cell pellet as described previously. After incubation and washing, the cell pellet was resuspended in 0.5 ml of Isoton II and flow cytometry was performed on a FACScan flow cytometer (BD) using prestored settings (Brotherick et al, 1995). Ten thousand cells (debris was excluded by threshold) were collected. Data analysis was performed using Lysis II software. Cytokeratin-positive or -negative cells were gated, and median fluorescence (PE) values determined from the FL2 histogram for SA-PE-stained (control) and ER-stained cells. Binding capacities were evaluated from the standardized QSC bead equation (QSC, Flow Cytometry Standards corporation, NC, USA) as previously described (Brotherick et al, 1995). Data were calculated as the number of oestrogen receptors (determined as the test sample minus the control level).

### Statistical analysis

Statistical analysis was performed using SPSS PC program to perform the Mann–Whitney *U*-test and generate 95% confidence intervals.

### RESULTS

MCF7 cells showed positive labelling with ER 1D5 and lymphocytes demonstrated only background levels of autofluorescence, confirming antibody specificity.

The levels of ER were measured on 40 tamoxifen-treated and 38 untreated patient breast cancers. Levels of ER were examined on the total cell population without using a gating protocol (Figure 1B). Using a cytokeratin gate (R1, Figure 1A) the level of ER was determined on the cytokeratin-positive population (Figure 1C). The percentage cytokeratin-positive cells was not less than 50% of the total cells run, and the average per cent cytokeratin-positive cells was 78%. Using a second gate (R2, Figure 1A) ER levels were determined on the cytokeratin-negative cell population (Figure 1D).

Examination of ER levels in the total cell population showed a median value of 37 540 (95% confidence interval 31 867–45 359, $n = 38$) ER per cell in the untreated population. In the tamoxifen-treated population, a mean value of 23 350 (95% confidence interval 18 534–29 391, $n = 40$) ER per cell was seen. The Mann–Whitney *U*-test showed a significantly lower level of ER expression in the tamoxifen-treated group ($P = 0.002$, Figure 2).

Examination of ER levels in the cytokeratin-positive cell population showed a median value of 46 000 (95% confidence interval 34 365–55 570, $n = 38$) ER per cell in the untreated population. In the tamoxifen-treated population, a mean value of 19 108 (95% confidence interval 18 990–34 196, $n = 40$) ER per cell was seen. The Mann–Whitney *U*-test showed a significantly lower level of ER expression in the tamoxifen-treated group ($P = 0.006$, Figure 2).

Examination of ER levels in the total cell population showed a median value of 22 647 (95% confidence interval 20 545–28 301, $n = 38$) ER per cell in the untreated population. In the tamoxifen-treated population, a median value of 20 381 (95% confidence interval 18 358–25 384, $n = 40$) ER per cell was seen. The Mann–Whitney *U*-test showed no significant difference in the level of ER expression in the tamoxifen group compared with the untreated group ($P = 0.4$, Figure 2).

### DISCUSSION

Oestrogen receptor status has been reported to be of prognostic value (Howell et al, 1984); however, more recently its value for prognosis and therapeutic response has been disputed (Maki and Hoehn, 1989) and the limitations of the oestrogen receptor assay reported (Poulsen, 1981). In the paper by Cohen et al (1988) image cytometry has been reported to allow both quantitation and examination of heterogeneous tumours. In our paper we report the use of ER 1D5 antibody in conjunction with an α-cytokeratin antibody to quantify the ER status of primary breast cancers by flow cytometry without the need for fixation and prolonged incubation (Brotherick et al, 1995).

The short-term treatment of breast cancer with tamoxifen has been reported to increase ER levels (Horwitz et al, 1978; Waseda et al, 1981). Although high ER levels are prognostically good, treatment with tamoxifen should block the oestrogen receptor.

© Cancer Research Campaign 1998

**Figure 1** Figure 1 illustrates the gates used to examine ER expression on cytokeratin-positive and cytokeratin-negative populations. **A** illustrates the cytokeratin-positive (R1) and -negative (R2) gates. **B** illustrates a histogram of fluorescence (ER) for the total cell population, **C** for the cytokeratin-positive gated cells and **D** for the cytokeratin-negative population.

Therefore, rises in ER levels in tamoxifen-treated patients preoperatively could elicit a stimulatory effect in non-responding cells, i.e. cause proliferation of phenotypes associated with poor prognosis. It would be useful to confirm ER status shortly before operation to determine if tamoxifen treatment has an effect in the 3-week period before operation and after diagnosis.

Long-term tamoxifen treatment decreases ER levels (Lacobelle et al, 1986), and indeed in the short term some findings confirm decreased ER levels (Montoya et al, 1992). Our findings concur with those of Montoya et al (1992), with significant falls in ER levels reported in those patients treated with tamoxifen 3 weeks before operation. Interestingly, some ER expression is seen in the cytokeratin-negative population and may reflect ER levels in fibroblasts. These levels are also seen to fall, although not significantly, with tamoxifen treatment. It may be important to examine this cell population as cytokeratin-negative tumour cells could theoretically be present. Logical progression of the research would be to compare pre- and post-tamoxifen samples from the same patient using fine-needle aspiration samples. Ethical permission and the need to perform an invasive procedure are points that will need addressing and were determining factors in this study when pre-tamoxifen sampling was not performed.

We conclude that use of flow cytometric analysis of ER is a rapid, reliable and quantifiable methodology that can be applied to monitoring receptor levels in pre-operative tamoxifen-treated patients.

© Cancer Research Campaign 1998 

**Figure 2** Figure 2 illustrates the number of ER molecules per cell in tamoxifen-treated or untreated breast cancer patients. Three groups have been compared: □, ER expression in the cytokeratin-positive population; ▨, cytokeratin-negative population; ⊠, in the total population (error bars = s.e.m., $n$ = 40 (tamoxifen+) and $n$ = 38 (tamoxifen–)

### ACKNOWLEDGEMENTS

The authors would like to thank Marianne Broe of DAKO A/S Denmark for the provision of conjugated ER-1D5, her cooperation throughout this study and her continued support. Mr C Charlton and Mrs T Johnson, Department of Histopathology for their help with sample collection. We would also like to thank the National Lotteries Charity Board and the Women's Cancer Detection Society (WCDS) Gateshead for their financial support.

### REFERENCES

Angus B, Purvis J, Stock D, Westley BR, Samson ACR, Routledge EG, Carpenter FH and Horne CHW (1987) A new monoclonal antibody recognising low molecular weight cytokeratins effective for immunohistochemistry using formalin fixed paraffin embedded tissue. *J Pathol* **153**: 377–384

Brotherick I, Lennard TWJ, Wilkinson SE, Cook S, Angus B and Shenton BK (1994) A flow cytometric method for the measurement of epidermal growth factor receptor: A comparison with the radio-ligand binding assay. *Cytometry*, **16**: 262–269

Brotherick I, Lennard TWJ, Cook S, Johnstone R, Angus B, Winthereik MP and Shenton BK (1995) Use of the biotinylated antibody DAKO-ER 1D5 to measure oestrogen receptor on cytokeratin positive cells obtained from primary breast cancer cells. *Cytometry* **20**: 74–80

Cohen O, Brugal G, Seigneurin D and Demongeot J (1988) Image cytometry of estrogen receptors in breast carcinomas. *Cytometry* **9**: 579–587

Early Breast Cancer Trialist's Collaborative Group (1992) Systemic treatment of early breast cancer by hormonal, cytotoxic, or immune therapy: 133 randomised trials involving 31 000 relapses and 24 000 deaths among 75 000 women. *Lancet* **339**: 1–15, 71–85

Horwitz KB, Koseki Y and McGuire WL (1978) Estrogen control of progesterone receptor in human cancer: role of estradiol and antiestrogen. *Endocrinology* **103**: 1742–1751

Howell A, Harland RNL and Bramwell VHC (1984) Steroid-hormone receptors and survival after first relapse in breast cancer. *Lancet* **i**: 588–591

Kumar V, Green S, Stack G, Berry M, Jin JR and Chambon P (1987). Functional domains of the human estrogen receptor. *Cell* **51**: 941–951

Lacobelle S, Scambia G, Forcucci-Zulli M and Gentiloni N (1986) Effects of tamoxifen on oestrogen and progesterone receptors in human breast cancer. *Rev Endo-Rel Cancer* (suppl.) **19**: 27–32

Maki HS and Hoehn JL (1989) Influence of oestrogen receptors on survival and recurrence in patients with breast cancer without lymph node metastases. *Arch Surg* **124**: 377–380

Montoya F, Barbazan MJ, Schneider J, Matorras R and Rodriguez-Escudero FJ (1992) Variations in estrogen and progesterone receptor levels after short term tamoxifen treatment in breast carcinoma. *Oncology* **49**: 422–425

Nomura Y, Tashiro M and Shinozuka K (1985) Changes of steroid hormone receptor content by chemotherapy and for endocrine therapy in advanced breast cancer. *Cancer* **55**: 546–551

Poulsen HS (1981) Estrogen receptor assays. Limitations of the method. *Eur J Cancer* **6**: 495–497

Richards MA, Smith IE and Dixon JM (1994) ABC of Breast Diseases. Role of systemic treatment for primary operable breast cancer. *Br Med J* **309**: 1363–1366

Sawka CA, Pritchard KI, Paterson AH, Paterson AHG, Sutherland DJA, Thomson DB, Shelley WF, Myers RE, Mobbs BG, Malkin A and Meakin W (1986) Role and mechanism of action of tamoxifen in premenopausal women with metastatic breast carcinoma. *Cancer Res* **46**: 3152–3156

Schutte B, Scheres HME, De Goij, AFPM, Rousch MJM, Blijham GH, Bosman FT and Ramaekers FCS (1992) Flow cytometric steroid receptor analysis. *Prog Histochem Cytochem* **26**: 68–76

Waseda N, Kato Y, Imura H and Kurata M (1981) Effects of tamoxifen on estrogen and progesterone receptors in human breast cancer. *Cancer Res* **41**: 1984–1988

Yamamoto KR and Alberts BM (1976) Steroid receptors: elements for modulation of eukaryotic transcription. *Annu Rev Biochem* **45**: 721–746

 © Cancer Research Campaign 1998